# Landmarks in wayfinding: a review of the existing literature

**DOI:** 10.1007/s10339-021-01012-x

**Published:** 2021-03-08

**Authors:** Demet Yesiltepe, Ruth Conroy Dalton, Ayse Ozbil Torun

**Affiliations:** 1 Department of Architecture and Built Environment, Northumbria University, Newcastle upon Tyne, UK; 2 School of Architecture, Lancaster University, Lancaster, UK

**Keywords:** Landmark, Wayfinding, Navigation, Literature

## Abstract

Landmarks are accepted as one of the vital elements in both virtual and real environments during wayfinding tasks. This paper provides an overview of the existing literature on the selection of landmarks in wayfinding mostly in large-scale urban environments and outdoors by discussing two main aspects of landmarks: visibility and salience. Environments and layouts used in previous studies, different tasks given to people and the main findings are explained and compared. Summary tables are created from these findings. The review concludes that there is mostly a consensus on the selection of landmarks, when considering their location. Accordingly, landmarks on route and also at decision points (with a turn) are more effective during wayfinding tasks. However, visibility of landmarks as well as visual and cognitive saliency need to be further investigated using different environments, tasks or different levels of familiarity with environments.

## Introduction

Wayfinding is goal-directed movement in which people aim to reach a destination that is not directly seen nor sensed from the origin (Montello 2005; Montello and Sas 2006). People know or learn the environment (spatial knowledge) to complete wayfinding tasks. As earlier studies mention, there are three components of spatial knowledge: landmark, route and survey knowledge (Siegel and White 1975). Accordingly, while exploring environments people notice and recall landmarks, learn a route to follow and they may also discover alternative routes. Hence, knowing an environment is closely related to knowing landmarks; their location, identity and interaction with their surroundings.

### Introduction to landmarks

Landmarks are one of the five elements of the built environment as identified by Lynch (1960). They are easily *identifiable*, and more likely to be selected as a significant *point of reference*. Lynch did not mention only the objects themselves, but also their relationship to their surroundings. Hence, according to the author, landmarks should *contrast with their background* or have a clear shape or another specific characteristic that makes them *prominent*. Moreover, he argued that an object can be recalled as a landmark by itself as well as it can be recalled as a landmark when it is part of a group. Hence, for example, a group of trees or high-rise buildings in an environment can be used as a reference point by people. On the other hand, the environment might change over time, which might affect the selection of landmarks or even the existence of landmarks. Richter and Winter (2014) mentioned that the first skyscrapers in Chicago were landmarks; however, many other skyscrapers were built over time, changing what people call a landmark.

The early definition made by Lynch (1960) is still one of the most significant definitions in the literature as it gives guidance to the characteristics of landmarks. Additional characteristics were also discussed in the literature. Richter and Winter (2014) stated that landmarks serve as anchor points and points of reference. The importance of the visual characteristics of landmarks, location of landmarks (Lovelace et al. 1999; Siegel and White 1975; Sorrows and Hirtle 1999) and their relationships with their environments, as prominent objects (Caduff and Timpf 2008), were discussed previously. It was also pointed out that the meaning of objects and their cultural, political or social impact on people might make them more *noticeable* (Caduff and Timpf 2008; Couclelis et al. 1987; Sorrows and Hirtle 1999). Couclelis et al. (1987), for example, identified landmarks as *distinctive* features or objects with *symbolic meanings*. Thus, these characteristics help landmarks be noticed and remembered (Presson and Montello 1988; Sadalla and Magel 1980). It was also stated that the brevity of a landmark description (the number of words used) is also significant (Burnett et al. 2001) since a landmark should not require a long explanation for specific tasks such as route description. It should be as precise and brief as possible (Nuhn and Timpf 2018). For instance, rather than saying “turn right after the stone building with a big entrance and high windows,” one could say “turn right after the bank.” This would be a shorter instruction pointing a specific function so that it would be easier to remember. The definition of landmarks usually points to the interaction between people and space as landmarks are selected as a result of this interaction. Another example for this relationship can be seen in Richter and Winter’s definition as they refer to landmarks as “geographic objects that structure human mental representations of space” (2013 p. 205). In this definition, it is mentioned that a space can be represented by a group of objects or their relationship in people’s mind. When we think about Amsterdam, for instance, we may initially think of the canals. Or for Venice, we might think of the bridges. These landmarks become one of the most significant representations of these cities. People’s interests, backgrounds and thoughts have an effect on their landmark selection.

### Research aim and methodology

In this review article, we aim to better understand how people choose landmarks to find their ways. It was claimed previously that any item in an environment can act as a “landmark” (Ishikawa and Nakamura 2012; Quesnot and Roche 2015a). In which case, how can we know what makes a landmark more preferable during wayfinding? Why do we select some landmarks over others? Is it due to the above-mentioned characteristics (e.g., the uniqueness of landmarks or contrast with the background) and if so, can we measure this? There are a great number of studies on characteristics of landmarks; however, there is not any current article where the research on selection of landmarks is reviewed. Therefore, in this study we would like to better understand the characteristics of landmarks that make them more significant than others in different environments.

The objectives of this study are: to understand the main findings of the literature on wayfinding and landmarks, to identify the consensuses and the gaps of the literature and to provide insights into future research. In the end of this review, we would like to answer two questions:

1. Which characteristics of landmarks make them more effective during a wayfinding task? What consensus exists in the literature?
2. What are the gaps in the literature? And what else can be done to better understand the relationship between environments, landmarks and people?

As the above-mentioned examples highlight, landmarks are closely related to the environment and people. Therefore, while analyzing landmarks, environment and people are also discussed. The review includes any possible step during wayfinding, such as route description, orientation or actual wayfinding. It mostly focuses on studies in large-scale urban environments and outdoors rather than indoor navigation or rural environments. Studies comparing different features of landmarks as well as studies on route knowledge are the main focus of this study (yet, papers on survey knowledge can be found here as well if they are closely related to the focus of the review). In addition, in this paper visual cues are investigated rather than olfactory or acoustic cues. On the other hand, papers on specific topics (e.g., diseases, animal-related research, research on specific age groups, people in different mood), studies only on autonomously navigating robot systems and self-movement cues are excluded in this research. The methodology and the findings of the papers are summarized in “Visibility of landmarks” and “Saliency of landmarks” sections, and the agreements and disagreements (gaps) are defined accordingly. These studies were categorized based on relevant tasks and measures of wayfinding, as discussed in the paper, and their findings relevant to the discussion on landmarks are summarized.

## Landmarks in wayfinding studies

When literature was analyzed, it was observed that landmarks can be used for different purposes, including identifying specific locations (Downs and Stea 2011), finding one`s way to a certain location (Klippel and Winter 2005), and orienting oneself in order to understand whether the selected path is correct (Michon and Denis 2001; Philbeck and O’Leary 2005). Landmarks help people organize their spatial knowledge and locate themselves with respect to a specific destination more easily (Couclelis et al. 1987). Most commonly, landmarks provide information for people to better understand when they should change their orientation along a route (Michon and Denis 2001). They are effective on route learning (Tlauka and Wilson 1994; Waller and Lippa 2007) and decision making (Golledge 1999). Therefore, landmarks can be used for various purposes at different stages of wayfinding. In this study, we focus on papers that aimed to explore the association between landmark usage and wayfinding.

The literature on landmarks in wayfinding can be divided into two headings: visibility of landmarks and saliency of landmarks. The visibility of landmarks is closely related to saliency of landmarks. However, since visibility is mentioned separately in various studies, we also chose to discuss this characteristic in a separate section. Moreover, a landmark has to be visible to be salient (an invisible landmark cannot be salient) but not all visible landmarks are also salient. This is also why we separated these two sections. Nevertheless, we believe that the reader will be able to follow the relationship between these concepts. Based on the categories we used in this paper and the brief definitions we have so far provided, our working definition of a landmark is: “*any salient object that is personal (so that it can be seen and used by someone while it is not used by someone else), communicable (so that it can be described easily),*^1^*and visible either from a distance or close up in an environment such that it can be used in the wayfinding process for various tasks (e.g., route definition, orientation* *etc.)*.”

## Visibility of landmarks

Depending on their visibility during a wayfinding process, landmarks can be divided into two categories: global and local landmarks (Yesiltepe et al. 2019). Distant objects such as mountains and towers, which can be observed from a great number of vantage points, are accepted as global landmarks (Steck and Mallot 2000). Lynch (1960) described global landmarks as elements seen from many angles and distances, and even those visible above smaller elements. In contrast to global landmarks, local landmarks are only visible from close up (Steck and Mallot 2000); they are visible only from a limited area and only from certain approach directions (Lynch 1960). Local landmarks can be trees, storefronts or signs (Lynch 1960) and they may be more personal (Dalton and Bafna 2003). A vast number of studies used this definition for global and local landmarks. In their study, Castelli et al. (2008 p. 1648) also made a similar definition: “global landmarks, *being potentially visible from any point within the navigational environment* and so from a great distance, become absolute points of reference, favoring orientation strategies in survey terms.” A similar definition was also made by Lin et al. (2012). At this point, a question arises from this definition: is it necessary for a landmark to be *visible from any point* in an environment? Is it not possible for a landmark to be visible from many locations in an environment—as Lynch mentioned—but not from all and yet still help people to have global orientation? From another perspective, if we try to apply this “visible from any point” idea to current cities, how many global landmarks can we define (i.e., those that are visible from everywhere)? If we accept that global landmarks are objects that are visible from multiple points in an environment, then a further question arises: how can we actually identify a threshold between a global and a local landmark? When does a landmark stop being local and become a global one? Is there a way to measure this? A clear definition and a threshold for distinguishing global landmarks from local ones are needed, especially for large-scale environments with multiple visual cues.

### Findings of the studies on visibility of landmarks

Even though there is a considerable body of research about the visibility of landmarks, their results vary. Numerous studies argued that the role of local landmarks on wayfinding is more accurate. Evans and colleagues (1984), for instance, focused on the effect of stress, landmarks and path configuration on environmental cognition. They used the terms “internal” and “external” landmarks and created four different environmental conditions: no landmarks on non-grid pattern, internal landmarks on non-grid pattern, external landmarks on non-grid pattern and no landmarks on a grid pattern. In their experiments, a realistic model of a fictitious urban area was designed, and the viewpoint of a moving automobile passenger was simulated. After watching the video of the simulation, all participants were asked to consider photographs of the environment and indicate their degree of confidence in recognizing each image (the images included the different landmark conditions). Internal landmarks were placed within the context of the setting while external landmarks were placed not in the immediate field but in the distant line of sight. Consequently, the researchers discovered no significant differences between the internal and external landmark conditions. However, they observed a trend for internal landmarks to be more helpful in recognition than external ones. The authors also highlighted that landmarks improved route learning more in the presence of a non-grid layout, suggesting that further research needed to be conducted in a variety of different layout types. In another study, Ruddle et al. (2011) hypothesized that adding both global and local landmarks to an environment would reduce the number of navigational errors people make. They designed four virtual marketplaces in a grid layout, and participants were asked to navigate in four different conditions: no landmarks, only local landmarks, only global landmarks and both local and global landmarks. All landmarks consisted of pictures; the positions of landmarks were automatically generated by a computer program. Participants were asked to find their way in the virtual environments (VE) and then complete a questionnaire in which they were asked to draw a map of the route they used and explain their turning decisions. The researchers observed that although local landmarks did reduce participants’ errors, global landmarks did not influence the overall number of errors. Moreover, local and global landmarks interfered with each other; hence, the researchers observed that participants, who were provided with both kinds of landmarks, made more errors compared to those provided with a single kind of landmark. In the second study, the authors compared the performances of people who physically walked through the VE and who traveled by physically turning but moving forward with a joystick. They discovered that participants who physically walked in the VE made fewer errors compared to others moving forward with a joystick.

Meilinger et al. (2015) aiming to explain the interaction between proprioceptive and global cues also compared two different global landmark information: global landmarks providing heading-only information (a mountain silhouette) and those providing both heading and distance information (a factory hall). They designed a virtual labyrinth layout and thirty-three participants were randomly assigned to one of three conditions: self-movement cues, self-movement and orientation cues, and finally self-movement, orientation and distance cues. During the learning phase participants walked within the environment; later in the test phase, they were asked to point to a target whose name appeared on a screen as accurately and quickly as possible. Results of this study showed that global landmarks did not have any dramatic influence on orientation. The researchers concluded that further research can be carried out with a higher number of participants and that different results might be observed in a linear environment as the participants might profit from global landmarks. Moreover, in their previous study, Meilinger, Riecke et al. (2014a, b) aimed to explain how the location of different spaces are represented in memory through global reference frames, multiple local reference frames and orientation-free representations, using two experiments. In the learning phase, participants were asked to walk five times through the VE (labyrinth), and in the test phase, they were asked to point to seven learned targets. Similar to previous research, the results of this study showed that participants relied on local reference frames rather than global reference frames or orientation-free representations. Other studies conducted in virtual environments also found no advantages of global landmarks on learning (Credé 2019). In another experiment, participants navigated four virtual environments with different landmark conditions: only global landmarks, only local landmarks, global and local landmarks and no landmark conditions (Gardony et al. 2011). Global landmarks were four distant, multistorey buildings whereas local landmarks were four different items: a rock, bush, wagon and haystack. Participants were asked to navigate and find invisible target points as quickly as possible. Results of this study also showed that local cues were perceived as the key information when the environment included both types of landmarks. It might still be important to understand the reasons why people make more errors in wayfinding tasks when they are provided with both types of landmarks, since this result was unexpected. These studies also demonstrated the impact of different layouts: it was mentioned that landmarks can be more effective when the environment is linear and when the layout is not gridded.

Another body of research emphasized the significance of familiarity to the environment. Lynch (1960) mentioned that people use local landmarks to find their way in an environment if they are not unfamiliar with the environment. In his study in real environments, Lynch made interviews with people and asked them to describe routes, draw sketches and recognize the location of cues. He observed that participants who are not familiar with the environment tend to use global landmarks. In another research that also supported Lynch’s findings, Kelsey (2009) aimed to define global and local landmarks and she also noted that there is no consensus on the definition and categorization of landmarks. Thus, she defined global and local landmarks based on the size and visibility of objects and tested these in three experiments in VEs organized using a rectangular grid layout. In the first experiment, Kelsey focused on the impact of landmark type (global/local) independent of their location; in the second one she focused on the location of landmarks (internal/periphery); and in the last experiment, she focused on the combined effect of these two factors. Participants were asked to complete a wayfinding task, draw a map and decide which way to go for a specific task. Similar to Lynch’s study, the findings of this research suggested that global landmarks were more effective in unfamiliar environments than local landmarks and when familiarity increased local landmarks started improving the wayfinding performance. The above-mentioned studies highlighted the significance of people’s familiarity with the environment as they recall global landmarks more when they are unfamiliar with the environment.

The idea that both global and local landmarks are effective on wayfinding performance was also argued by different researchers. In their study, Steck and Mallot (2000) defined global landmarks as compasses. They hypothesized that people might use different strategies; they might rely only on local landmarks or on global landmarks, or they might use different landmarks at different locations, or they might use both kinds of landmarks in combination. After creating a virtual environment consisting of a regular hexagonal grid of streets, the researchers defined intersections using local landmarks (e.g., a phone box) and added global landmarks (e.g., television tower) to the environment. After two training phases, where participants became familiarized with the environment, they were transported to one of the decision points and asked to make a turn for a specific goal point. Their movement decisions were recorded by the researchers. It was observed that some participants used only local landmarks for their decisions while others relied only on global landmarks. In addition, some participants used local landmarks at one position and global landmarks at another. As a result of these experiments, the authors asked a new question: “how do participants select a landmark for a specific decision?”. The researchers discovered that at locations where participants preferred local landmarks, landmarks had different functions or visual characteristics (they were objects such as a gas station or a tower). In addition, global landmarks were less visible at these points as they were occluded by trees. Therefore, they argued that to understand the selection process of landmarks, not only a visibility classification—as global and local landmarks—but additional information (saliency) should be analyzed. Moreover, Schwering et al. (2013) attempted to determine the types of information that support orientation during wayfinding. They first asked participants to draw sketch maps for first-time visitors from one direction while another group was asked to draw sketch maps from the reverse direction (a real-environment task). Then both groups were asked to provide verbal instructions for the routes they drew. In addition, the researchers aimed to compare machine-generated route instructions with humans’ route instructions. The findings of the study showed that machine-generated instructions used only street names and distances while people used landmarks. They discovered that in both tasks (verbal instructions and sketch maps), participants provided both global and local information to help with orientation. Furthermore, the authors also noted that sketch maps indicate global orientation more while verbal instructions convey local orientation. In another study, Schwering et al. (2017) aimed to explore the usage of global and local landmarks in wayfinding instructions with a larger dataset and more routes. For this purpose, they chose routes starting from outside, and ending inside a city center (global landmarks, according to their definition) or starting outside a city center, crossing it and ending up outside it or passing by several cities with city centers. Three experiments were conducted in this study and participants were asked to describe a familiar area with both sketch maps and verbal instructions, point to specific locations and walk a pre-defined route with a smart phone. Participants were tested on three different routes and there were at least two global landmarks on each route. Results of the study showed that local landmarks were the most commonly mentioned spatial feature in both route descriptions and sketch maps; however, all participants included global landmarks in their descriptions as well. In addition, global landmarks were drawn on sketch maps more often. The researchers concluded the study by saying that both global and local landmarks along the route were used for orientation.

Similarly, Schwering, Li and colleagues (2014) aimed to explore the use of landmarks in verbal descriptions for routes at different scales and through different transportation modes (cycling and driving). The researchers described three routes with different scales: the first route was the shortest (1.2 km), the second route covered the city and was approximately 5 km, and the last route was at an environmental scale (approximately 18 km). All participants were given a laptop and asked to write down the verbal route instructions for to someone unfamiliar with the study area. While analyzing the global and local landmarks, the researchers focused on landmarks along the route or at potential or actual decision points for local landmarks. They focused on point-based landmarks (e.g., buildings) and regional landmarks (e.g., city centers) for global landmarks. It was found that both local and global landmarks were used regardless of scales. The findings of this study suggest that local landmarks are mostly used in verbal descriptions whereas global landmarks are used in wayfinding and spatial orientation in large-scale environments. The researchers concluded the study by mentioning that they only used one task (verbal descriptions) and further research could be conducted by including additional tasks, such as sketch maps and comparing the results. Finally, they suggested that using landmarks, not only at decision points but also along routes, can make route orientation more efficient. Moreover, in another study aiming to clarify the effect of different verbal instructions, Li et al. (2014a) concluded that wayfinding and spatial orientation can be achieved by using both global and local landmarks. They conducted the research in Germany and used verbal instructions consisting of landmarks at decision points, on-route landmarks and distant landmarks (for maintaining orientation). Participants were instructed by machine-generated, skeletal or orientation-based instructions. They were asked to draw sketch maps and estimate directions and distances at various locations. The researchers discovered that machine-generated instructions were less effective for acquiring spatial knowledge. They also argued that the efficiency of a wayfinding task can be achieved by including both types of landmarks at various locations. Finally, in another study, which aimed to determine the hierarchy of landmarks (Anacta et al. 2014), both global and local landmarks were used and global landmarks were categorized as point or regional features. Point-like landmarks referred to specific buildings whereas regional landmarks referred to landmarks with an areal extent (e.g., mountains). Local landmarks were categorized as landmarks along a route and landmarks at decision points with-a-turn. This study was also conducted in Germany. Participants were asked to provide wayfinding instructions for someone unfamiliar with the city through verbal descriptions and a sketch map. They used both global and local landmarks for route descriptions. All these studies demonstrated that both global and local landmarks can help people during a wayfinding task. However, it should be stated that landmark use is task-related since the above-mentioned studies showed that, depending on a task, one type of landmark tends to be used more frequently.

Finally, the last group of studies suggests that global landmarks are more effective during wayfinding. In Lin et al.’s study (2012), global landmarks were defined as objects that can be seen from everywhere inside a maze (e.g., a tower, lighthouse, windmill), whereas local landmarks were located on walls (cubic blocks)—visible from only one side of the blocks (e.g., a fish, banana, bird)—inside the grid maze. After completing the learning period, the participants were asked to navigate the environment and find a specific target picture. Once they found one picture, they were moved to a random location and asked to find another target picture. The researchers discovered that participants traveled longer paths in the local landmark condition compared to the global landmark condition. They explained these results through the anchor-point usage of global landmarks for orientation and the time participants spent to find the local landmarks. Hence, this study is essential as it emphasizes the importance of global landmarks within virtual environments. Li et al. (2016) suggested that the existence of global landmarks might help people orient themselves between locations. They conducted three experiments (depending on the findings of the first experiment, they designed augmented reality models for the other two experiments). In the first experiment, four two-storey virtual buildings with the same layout complexity were used. They defined two types of global landmarks: an outdoor, global landmark (a church visible from the building’s windows) and an indoor, global landmark (a statue in an atrium) visible from multiple locations. Participants completed ‘pointing’ (turning to face a target point), ‘wayfinding’ (finding a target point by the shortest route) and ‘drilling’ (on arrival, stating which object/room was directly above/below them) tasks. Data showed that increasing visual access to both indoor and outdoor global landmarks significantly promoted users’ cognitive map development (please see Tables 1 and 2 for the summary of the mentioned papers).

**Table 1:** Summary table of the studies on visibility of landmarks. The names are listed based on their appearance in the text

Author	Year	No. of people	Environment		Layout		Task						Saliency			Visibility		Finding	Overall
			Real	Virtual	Grid	Non-grid	Wayfinding	Orientation	Route description	Recognition /preference	Sketch	Other	Visual	Structural	Cognitive	Global	Local		
Evans et al.	1984	128		X	X	X				X	X					X	X	No significant differences between two landmark conditions. But a trend was observed for internal landmarks to be more helpful in recognition than external ones.	Local landmarks
Ruddle et al.	2011	63–44		X	X		X			X	X					X	X	Local landmarks reduced participants’ errors; however, global landmarks did not influence the overall number of errors.	
Meilinger et al.	2015	33		X		X		X								X		Participants did not profit much from additional global landmarks when bodily-self-movement cues were available.	
Meilinger, Riecke et al.	2014	18–20		X		X		X								X	X	Participants relied on local reference frames rather than global reference frames or orientation free representations.	
Credé et al.	2019	48		X		X	X	X		X						X	X	No advantage of global landmark configurations was observed for survey knowledge acquisition.	
Gardony et al.	2011	48		X		X	X									X	X	An overall preference was observed for local cues. Local landmarks were perceived as central information.

**Table 2:** The rest of the summary table of the studies on visibility of landmarks

Author	Year	No. of people	Environment		Layout		Task						Saliency			Visibility		Finding	Overall
			Real	Virtual	Grid	Non-grid	Wayfinding	Orientation	Route description	Recognition /preference	Sketch	Other	Visual	Structural	Cognitive	Global	Local		
Lynch	1960	60	X		X	X			X	X	X		X	X	X	X	X	It was stated that people familiar with the environment rely on local landmarks more whereas people unfamiliar with the environment rely on global landmarks	Global and local landmarks
Kelsey	2009	60–87–78		X	X		X			X	X					X	X	It was stated that people familiar with the environment rely on local landmarks more whereas people unfamiliar with the environment rely on global landmarks	
Steck and Mallot	2000	32–36		X	X (hexagonal grid)			X		X						X	X	Some of the participants relied on global landmarks; others relied on local landmarks or both global and local landmarks	
Schwering et al	2013	18	X			X			X		X					X	X	People used local landmarks most commonly in two task; however, global and local landmarks were provided by people to help maintain orientation	
Schwering et al	2017	30–21–60	X			X	X	X	X		X					X	X	Both global and local landmarks were included in the instructions and used for orientation. Global landmarks were drawn on sketch maps more often	
Schwering, Li et al	2014	21	X			X			X							X	X	Both global and local landmarks were used in verbal descriptions. Local landmarks were mostly used in verbal descriptions whereas global landmarks were used for orientation	
Li, Fuest et al	2014	11	X			X		X	X		X					X	X	Including both global and local landmarks in route instructions contributed to orientation and cognitive mapping	
Anacta et al	2014	17	X			X			X		X					X	X	Both types of landmarks were used in giving route descriptions	
Lin et al	2012	30		X	X		X									X	X	Participants travelled longer paths in the local landmark environment than they did in the global landmark environment	Global landmarks
Li et al	2016	16–16–16		X		X	X	X								X	X	Increasing visual access to a global landmark through direct access promoted users’ development of a multi-level cognitive map significantly	
Li, Korda et al	2014	24	X			X	X	X		X	X					X	X	Including distant landmarks contributed positively in helping spatially orient people with low sense of direction	

In another study, Li et al. (2014b) presented a mobile map display (on mobile devices) that visualized distant landmarks at the edge of the mobile screen to support spatial orientation. They selected 13 landmarks in Münster (Germany) to implement in the mobile device and two versions of maps were used: with and without distant landmarks. People, who were unfamiliar with the study area, visited various locations in the area and used mobile devices to help their spatial orientation. They were asked to estimate their direction and distance from the start point three times during the experiment, and after completing the task, were asked to perform a landmark recall task. This research showed that global landmarks helped people to orient themselves. This subset of landmark research, above, is important for showing the significance of global landmarks in wayfinding tasks.

### Summary of the visibility of landmarks

In this section, first, we discussed the definitions of global and local landmarks. Do global landmarks need to be seen from multiple points in an environment, or should they be seen from everywhere? Previous studies focused on objects that can easily be identified as global or local landmarks (e.g., towers and mountains, constantly visible, for global landmarks). However, in the built environment the distinction is not always clear. Hence, we believe further research needs to be conducted to distinguish global landmarks from local landmarks and to identify a threshold between the two.

Second, despite numerous studies focus on global and local landmarks, they point to different findings. The effects of global landmarks, local landmarks and both global and local landmarks on wayfinding performance were identified by different papers. As Lynch (1960) and Kelsey (2009) stated previously, differences in landmark preference might be explained by environment-familiarity. However, Gardony et al. (2011) also pointed out that people rely upon local cues when the environment is smaller whereas they use global landmarks when the environment is large-scale. The scale of environments, which was discussed by several other papers (Learmonth et al. 2001; Smith et al. 2008; Weng et al. 2017), should therefore also be considered an important factor. In addition, the environment-layout was also highlighted as an important element in the usage of different types of landmarks (Evans et al. 1984; Meilinger et al. 2015). Hence, further research, which includes multi-scaled environments with varying layouts and participants with differing levels of environment-familiarity, needs to be undertaken. This would lead to a better understanding of the impact of different layouts, scales and different levels of familiarity on wayfinding. In addition, various studies have been conducted in virtual or real environments with differing, sometimes contradictory results. For instance, some studies in both real and virtual environments found differing use of local and global landmarks by people with varying levels of familiarity with an area.

Another important point emerging from the above-mentioned studies was the effect of the specific task on landmark usage. Results of the reviewed literature suggested that local landmarks are mostly used in verbal descriptions while global landmarks are used in sketch maps or in spatial orientation. This is an important finding since it shows the impact of navigational tasks on landmark preference. However, more research still needs be undertaken on the impact of landmark visibility on wayfinding. In addition, some studies on route description tasks compared machine-generated instructions with human-based and/or skeletal instructions. The results of these studies focused on the limitations of machine-generated instructions. However, more research still needs to be conducted on different route descriptions and their impact on people’s wayfinding performances.

Finally, studies on the visibility of landmarks demonstrated that global or local landmarks are influenced by other characteristics of landmarks (e.g., visual or semantic characteristics). For example, studies showing that a local landmark can be used by more people if it has a specific function or characteristic. Therefore, the visibility of landmarks cannot be considered as being independent of the visual, structural or cognitive characteristics of landmarks, which leads us to the next section.

## Saliency of landmarks

Another important theme discussed by researchers is the saliency of landmarks. Caduff and Timpf (2008) defined the concept of saliency as an object’s property if distinct, prominent or obvious compared to its surroundings. They argued that a landmark must be perceptually salient, in some sense, and it must contrast with its surroundings. Similarly, Hamburger and Röser (2014) stated that landmark salience is about those properties of an object that make it stand out from its surroundings. Götze and Boye (2016) argued that people choose salient landmarks since they are easily recognizable and memorable.

Different definitions were developed for the salient landmarks. One of the key contributions to the landmark saliency was made by Sorrows and Hirtle (1999). They identified three different landmarks for both real and virtual spaces: visual, cognitive and structural landmarks. According to their definition, a visual landmark is a physically prominent object (due to its shape, color, size etc.); a cognitive—or semantic (Klippel and Winter 2005)—landmark is related to the meaning of an object (i.e., historical/cultural meanings or widespread knowledge of the object), whereas a structural landmark is related to the importance of the object’s location. Visual landmarks contrast with their surroundings and, hence, become memorable. A differently colored building, a higher-sized building or an unusually shaped building might be *visually salient.* Cognitive landmarks, on the other hand, might be culturally or historically important and as such they have significance. Cognitive landmarks can be more personal, and people may miss them if they are unfamiliar with the environment. Finally, structural landmarks are typically in prominent location in an environment and thus are highly accessible (Sorrows and Hirtle 1999). They can be objects in a highly frequented location, or even an intersection itself, which are known (and possibly named) by people (Yesiltepe et al. 2020b). This three-tiered definition of saliency is essential as it covers different aspects of saliency. However, it also poses a challenge: how can we measure landmark salience using these criteria? Even though the cognitive aspect is defined as the cultural or historical effect of an object, how can we measure it? As Richter and Winter (2014) mentioned, the experiences of people can vary in different spaces or at different times. A place which lacks any visual or structural significance can have meaning for an individual with memories of this place. Thus, familiarity is a key factor for cognitive saliency. In addition, it is also important to understand how visual or structural saliency can be measured and compared for different landmarks.

The definition of saliency was further refined by other researchers. Burnett (1998), in his study on car navigation, aimed to understand the salient characteristics of landmarks. He pointed out three factors that can be significant for the wayfinding process: location (whether the position of a landmark allows identification), visibility (the ability to see a landmark) and uniqueness (the likelihood of a landmark being mistaken for other objects) of landmarks. The permanence of a landmark (always found at a specific location) is considered a prerequisite factor. Visibility relates to Sorrow and Hirtle’s “visual saliency” and location relates to “structural saliency.” Another key study by Caduff and Timpf (2008) claimed that both Sorrow and Hirtle’s (1999) and Burnett’s (1998) studies failed to characterize landmarks quantitatively and provide methods for assessing landmark salience for wayfinding. They introduced three terms of saliency: perceptual, cognitive and contextual. Similar to Sorrow and Hirtle’s definition, they identify the physical characteristics of objects for describing perceptual salience. However, they extended the definition by describing three categories of perceptual salience: location-based (color, intensity, texture orientation), object-based (size, shape and object orientation) and scene-context (topology and metric refinements). In addition to these definitions, observers’ experiences and knowledge are considered affective factors in defining salience. The researchers identified two components for cognitive salience: the degree of recognition (indicating how well objects can be identified from others) and idiosyncratic relevance (the personal importance of objects for observers). For the final component, contextual saliency, they focused on two types of contexts: task-based context (which includes the types of tasks) and modality-based context, which includes the mode of transportation and the number of resources.

Recently, Von Stülpnagel and Frankenstein (2015) examined how landmarks’ configurational salience impacts people’s perception compared to their visual salience. The configurational salience was measured through the use of visibility graph analysis (VGA).^2^ Landmark size (the number of cells it occupied), isovist size (number of cells from which a landmark was fully or partially visible) and integration (average visual distance to all cells) were calculated to measure a landmark’s configurational salience. Visual salience was rated by five raters using a 5-point Likert scale. The researchers observed that people tend to choose landmarks not only based on their visual characteristics but also on their configurational properties. Thus, we can argue that in addition to other components of salience, configurational characteristics of landmarks are also fundamental in identifying landmark salience. This definition was another important contribution to the saliency literature as it explored saliency of landmarks by using specific VGA analysis. However, this definition relates to structural landmarks, as it is dependent on the location of landmarks in an environment. This study was effective as it shows alternatives to measuring salience using VGA analysis.

### Measures used in explaining saliency

Many models and measures were later developed to identify salient landmarks and to analyze the visual, structural or cognitive (semantic) landmarks objectively. These models proposed alternative measures to calculate landmark saliency and focused on either the object itself, or people’s evaluations.

Raubal and Winter (2002) proposed that “semantic” salience could refer to the cognitive characteristics of landmarks, arguing that landmark saliency consists of cultural and historical characteristics of landmarks and explicit marks. In this non-experimental study, they focused on visual, semantic and structural characteristics of landmarks, and in order to analyze visual characteristics they used façade area (width x height of buildings), color (RGB color chart is used and checked to see if the color is different from the surrounding), shape (proportion of height of a building to its width) and visibility (2-dimentional area of the space covered by the visibility cone of the front side of a landmark). For semantic salience, historic or cultural importance of objects was examined (true–false questions) by using a database for the city of Vienna, Austria. If an object mentioned was in this database, they reported it as “true.” Explicit landmarks were defined as signs of buildings. So, for instance, if a building had a sign indicating the function of the building, then it was expected that this information would be mentioned in a route-description. Therefore, this information was also coded as “true” or “false.” Nodes and boundaries were used to describe structural salience. Hence, all characteristics mentioned by Sorrow and Hirtle were identified with measureable properties. This was an important contribution to the literature. Moreover, the concept of “visibility,” which is also significant for saliency of landmarks, was also included in this study. Hence, the researchers argued that if an object is more visible than any other object around it, it might be selected by more people (visual saliency).

Nothegger et al. (2004), who measured saliency by observing the visual and semantic characteristics of objects, extended Raubal and Winter’s work by proposing that orthoimages can be used to calculate the area of different shaped structures in order to define visual saliency. They analyzed visual and semantic salience with similar measures used by Raubal and Winter (2002) and calculated overall saliency for n*i*ne intersections along a route. The researchers compared those results with the results of human subject test. They organized a web-based questionnaire, showed panoramic images to people and asked them to rate the most prominent façade. The results of the study showed a significant correlation between the saliency model and participants’ answers. With these two studies, the authors suggested objective ways to measure saliency. However, the researchers claimed that it would be useful to apply these measures to larger datasets to identify the performance and the cost, which is also an important issue to explore further. In addition, it is also important to have reproducible models. In these models, even though the authors suggested various ways to measure landmarks objectively, it might be challenging to conduct the analysis for larger-scaled areas.

Winter et al. (2008) presented another model to build hierarchies of landmarks from saliency in another non-experimental study. They started with an assumption that any location in an environment can be described with references to landmarks; so, any point in a Euclidean map is in at least one landmark’s reference region. Therefore, the researchers claimed that environments can be defined either by hierarchic partitions of space, after which the most salient landmarks can be identified; or salient landmarks can be found first and then the partitions can be searched for. In this research, authors first defined the salient landmarks. They analyzed Hannover, Germany by focusing on junctions and using the buildings around the junctions to identify salient landmarks. Once the salient landmarks were clarified, they were considered as voronoi seeds and voronoi maps were created accordingly. Hence, each voronoi region was described by a salient landmark, which could help people to find their way or generate route descriptions. This model was important as it made clear connections with the environment by representing different urban spaces with specific landmarks. If a whole settlement can be defined with salient landmarks, this would allow people to find their way easier and not to get lost; but it also can help environments to be more attractive (landmarks with different shapes, color etc. can attract more attention). However, rather than focusing only on one building and its saliency scores, different landmarks could be compared, and more salient ones could be used in this study.

There is a plethora of research on the automatic selection of landmarks (Elias 2003; Elias and Brenner 2005; Kattenbeck, 2016; Kattenbeck et al. 2018; Lazem and Sheta 2005; Peters et al. 2010; Richter 2007; Tezuka and Tanaka 2005; Wither et al. 2013). Elias (2003) focused on building databases and categorized buildings based on their attributes in a non-experimental study (land use, size, number of immediate neighbors and orientation with the road, distance from road and height). Rather than focusing on a random point in an environment, Elias focused on landmarks at decision points. She used two algorithms to capture salience and mentioned that the results were promising as it was possible to identify salient objects. In another study, Elias and Brenner (2005) first identified landmarks by using the building information of the digital cadastral map of Germany. As such, they created a table with the information of semantics and geometry of the data in their non-experimental study. Similar to previous research (Elias 2003), they focused on specific attributes such as building use, building area, orientation to street. Later, they narrowed their selection by considering the positions of landmarks relative to the route and their visibility (the visibility of landmarks at decision points as well as visibility of landmarks while approaching decision points). In this work, in which they extended their previous research by using visibility, the researchers could define a landmark at each decision point. However, because the algorithm detected only one landmark at each decision point, it was not possible to detect other-potential landmarks. In addition, the authors claimed that they computed visibility for a single view. However, for a wayfinding task it is crucial to understand the environment from many angles and different points. Hence, rather than a specific point, the visibility of landmarks should be calculated along the entire route to have more accurate results. These two studies conducted by Elias and colleagues were important to automatically select salient buildings, and the database they used might be reproduced easier than previous auto-selection alternatives. However, it should be noted that in these latter alternatives, both landmarks (only buildings) and the saliency criteria used (only visual and semantic) were limited in their scope.

Lazem and Sheta (2005) identified landmarks (again, by using buildings) and created an attribute table including building height and width, color, building activity (for semantic saliency) and building order in the street (for structural saliency). They simulated three virtual cities with grid layouts, but the number of urban blocks varied in each city. In addition, they distributed different types of landmarks realistically by using a GIS dataset belonging to Egyptian cities. They observed that about 80%, 78% and 45% of objects in these three VEs were identified as landmarks, respectively, and the highest scored landmarks were governmental buildings, institutions and other buildings with specific functions. Hence, they claimed that this selection method was useful to detect landmarks. This study was important as it aimed to analyze three components of saliency and to adapt the results to three simulated cities. However, structural saliency and semantic saliency were analyzed through limited measures, which is a limitation of this work. Duckham et al. (2010) approached saliency of landmarks in a different manner; rather than using an actual landmark, they focused on a group of landmarks (class-level information). They developed a weighting system based on landmark categories (e.g., hotels, parks etc.) and measured the visual, semantic and structural characteristics of these categories. For visual saliency, they used physical size, prominence, proximity to road and differentiation from surroundings (for both day and night time). For structural saliency, they focused on spatial extent and permanence. Semantic saliency was based on ubiquity and familiarity with the environment as well as the length of descriptions. A scoring system was developed, and each category was ranked by a group of experts. This approach was then adapted for different routes in Melbourne, Australia. The researchers used the ‘Whereis’ national web-based navigation service, and points of interest (POI) defined on this web page were considered as potential landmarks. Then landmarks were eliminated based on their accessibility, the mode of traveling of people and cognitive salience of landmarks. The researchers concluded the study by arguing that landmark selection was both “direction dependent” and “route dependent”. As it would be easier to produce a dataset from categories, rather than individual landmarks, this alternative methodology can be meaningful, and it can be reproduced easily for different places at different scales. However, the results would not be as specific as individual landmark studies, which can be a limitation of this research.

Models were also developed through the use of different technologies. For example, a mobile application was developed by Wolfensberger and Richter (2015), who aimed to provide a tool for the manual selection of landmarks in which people can take pictures while their position can be detected via GPS. By using the GPS data, the visible area was scanned for possible landmark candidates. Once the landmark candidates were selected, their visual and semantic characteristics were quantified. Size (area) and visibility (distance and azimuth deviation to the user) of landmarks were used for visual characteristics; type (tags that describe the function such as shop, leisure) and cultural and historical significance (the number of tags and background information either from the object’s web page or from Wikipedia) were used for semantic characteristics. The application was then tested in both an urban and a rural area as well as by a naïve user. Thirty landmarks were collected in the urban area, and 10 landmarks were collected in the rural area through the application. The naïve user was then asked to capture the same landmarks, and 87% and 45% of the landmarks were found in urban and rural areas, respectively. The results of this study showed that the application worked reliably, and no significant problem occurred for the naïve user test. They concluded that a widespread use of the application was possible. This research is promising in many aspects: both visual and semantic characteristics were used, people were asked to actively participate in the study (they took pictures and defined landmarks), the application was tested in both urban and rural areas as well as by a naïve user. One issue the authors discovered was related to landmark candidates. Open Street Map (OSM) data were used for the study and the researchers excluded the landmarks that were not defined in OSM. In another non-experimental study, Quesnot and Roche focused on semantic salience and argued that Social Location Sharing datasets could serve as reliable sources for measuring semantic salience and determining automatic landmark selection (Quesnot and Roche 2015a). The researchers mentioned that user generated place databases provided information on users’ interests as well as the different uses of places (daytime or night-time), while providing information not only for buildings but also for other objects, such as parks or mountains, etc., which makes a database more significant. They claimed that the information can be collected from social media services (e.g., Facebook, Swarm etc.) and adapted to the automatic landmark detection systems by using uniqueness of venues and geosocial activity of venues. Five popular places and five intersections located in Paris and Vienna were selected, respectively, and the geosocial activity of these locations was explored. As a result, the researchers observed that historically or culturally significant places did not necessarily generate the highest activities on social media. Hence, the researchers stated that computing cognitive saliency based on historical and cultural significance of objects is not always sufficient. As an example, they mentioned that a café like Starbucks can be semantically more salient than a historical column. They also argued that cognitive saliency is related with two factors: travelers’ profile (their familiarity with the environment) and the intensity of the cognitive landmark. This research was also essential as it provided an alternative way of measuring salience, which might help researchers to better understand the underlying reasons of selecting different objects as landmarks (please see Table 3 for the summary of the mentioned papers).

**Table 3:** Summary table of the studies on the selection of landmarks. The names are listed based on their appearance in the text

Author	Year	No. of people	Environment		Layout		Task						Saliency			Visibility		Finding
			Real	Virtual	Grid	Non-grid	Wayfinding	Orientation	Route description	Recognition /preference	Sketch	Other (gaze etc.)	Visual	Structural	Cognitive	Global	Local	
Raubal and Winter	2002		X				Not an experimental study (method is adapted to an area only)						X	X	X			The most significant landmark at each decision point could be identified using all three characteristics of landmarks
Nothegger et al	2004	40	X						X				X		X			Computed total saliency was significantly correlated with subjects' evaluations (r = 0.58, p < 0.001)
Winter et al	2008		X				Not an experimental study (method is adapted to an area only)						X	X	X			A landmark could be defined for each area using visual and cognitive saliency
Elias	2003	Not an experimental study											X	X	X			By using a building dataset, researchers discovered that the suggested model helped to identify salient landmarks at decision points
Elias and Brenner	2005		X				Not an experimental study (method is adapted to an area only)						X	X	X			One object could be detected for each decision point
Lazem and Sheta	2005			X	X		Not an experimental study (method is adapted to an area only)						X	X	X			The algorithm used in this study helped researchers to successfully identify landmarks (factories, governmental buildings etc.)
Duckham et al	2010		X						X (experts)				X	X	X			Landmark candidates were detected. A way of adapting cognitive principles into navigation services is proposed
Wolfensberger and Richter	2015	1	X						X				X		X			87% and 45% of the landmarks detected by the application were found in urban and rural areas respectively by the naive user
Quesnot and Roche	2015a		X				Not an experimental study (method is adapted to an area only)								X			Historically or culturally significant places did not necessarily generate the highest activities on social media. The researchers stated that global cognitive landmarks should be added to route instructions if we do not have access to each traveler's spatial knowledge

There are also studies that focus on specific characteristics of saliency. In the next sub-sections, studies on location of landmarks (structural saliency), personal and emotional landmarks (cognitive saliency) and gaze behavior (visual saliency) are discussed.

#### Location of landmarks

The location of landmarks (in other words, structural saliency) can be discussed individually since various papers only focused on this topic. Structural saliency might be the area of research where there is greatest consensus. Chan et al. (2012) focused on the function of landmarks to understand how they help people in the wayfinding process. They explored the function of visual location information and presented a four-part taxonomy in a non-experimental study: beacons, orientation cues, associative cues and reference frames. According to this definition, the beacon landmark type includes single objects that point to the exact location of a goal location; orientation cues are visual cues that provide a heading direction; associative cues are single objects that give relevant information; and reference frames provide a framework for spatial encoding. Therefore, by using the location of a landmark, one can understand several aspects of a wayfinding task. Moreover, if the cues are effective (e.g., if they can be easily seen, etc.), then it is hypothesized that it would be easier to complete the task successfully.

A number of studies emphasized that landmarks located at decision points are more effective (Lynch, 1960) and are better-remembered (Aginsky et al. 1997; Janzen 2006; Janzen and van Turennout 2004). Janzen (2006), for instance, organized three experiments using recognition tasks in a VE with landmarks located at decision points and non-decision points. Participants first saw a film and then they were asked about objects they saw in the film and were instructed to draw a map of the environment including the landmarks. Results indicated that objects at decision points were recognized faster. Miller and Carlson (2011) also devised two experiments in which subjects learned a route through a virtual, artificial museum. They discovered that both objects at decision points with a turn and without a turn were recognized by people. Other studies also suggested that landmarks at decision points with a turn are remembered easier (Meilinger et al. 2014a, b; Meilinger et al. 2012). It is also argued that the main role of landmarks at decision points is to confirm one’s orientation/heading (Schwering et al. 2013) to understand whether a change in trajectory is needed to find the goal (Michon and Denis 2001). This makes landmarks at decision points more significant during wayfinding.

Klippel and Winter (2005) referred to Sorrow and Hirtle’s definitions of visual, cognitive and structural landmarks and argued that there was a gap in this research as structural landmarks were not defined objectively. In addition to the above-discussed location of landmarks, the researchers argued that structural properties should be countable and constant. In their non-experimental research, Klippel and Winter approached structural salience of landmarks by considering the position of landmarks along a route (e.g., on-route/off-route landmark, at dp or not at dp) and developed a taxonomy of structural landmarks. Accordingly, landmarks can occur at some distance from the route or somewhere along the route. If they are along the route, they can be located at decision points or between decision points. Similar to other papers, they also mentioned two options for landmarks at decision points: either with a direction change or with no direction changes. Moreover, they focused on landmarks at decision points (with direction changes), identifying three different conditions: landmarks passed before re-orientation, landmarks not passed (re-orientation without passing the landmark), and landmarks passed after orientation (landmarks that can be observed immediately after a turn). The different locations of objects then were used for calculating an overall value for landmarks. Landmarks on-route and at decision points got higher scores. Röser et al. (2012) also indicated the importance of structural salience. They conducted a study in a virtual, gridded environment, which they called Squareland. In the first experiment, participants were instructed to learn a route from a start to a goal location and then were asked where a landmark should be located (allocentric view). In the second experiment, they used egocentric perspective, considered visibility and 12 landmarks were located in the environment. Participants were asked to memorize a route and point the location of specific landmarks. Findings of this study also supported those of Klippel and Winter (2005). The researchers stated that the landmarks should be located on the side of the intersection on which a turn needs to be executed.

Claramunt and Winter (2007) also focused on the structural salience of objects and they used Lynch’s definition of the components of legible cities (Lynch 1960) to explain the structural salience of landmarks with four components: nodes, paths, barriers and districts. Nodes are counted by using the number of connected places. Paths are analyzed according to the links among places; thus, if places (or nodes) are directly linked to each other by one segment/line, they are considered as related. Places that have more links are considered as more salient. Barriers describe spaces that are resistant to cross (like urban blocks) and their connection with streets is thought as another measure. Finally, districts are accepted as either a single graph or clusters of nodes. As this study makes a connection with the components of settlements (Lynch 1960) and presents a detailed explanation of how to measure structural salience, it can also be considered an important non-experimental work in this field.

Not only landmarks at decision points but also on-route landmarks were discussed in past research. Lovelace et al. (1999) used landmarks at decision points as well as different criteria in their study in which they aimed to explore the effect of different locations on people’s wayfinding performance in familiar and unfamiliar environments. They used four different landmark conditions: choice point landmarks (landmarks on-route also at-a-turn), potential choice point landmarks (landmarks on-route, but not at-a-turn), on-route landmarks (landmarks on-route but not at a decision point), and off-route landmarks in a campus area. People were asked to give route directions, retrace their route and remember whether or not they saw a scene while traveling. The researchers discovered that landmarks on-route, but not specifically at decision points, were used for familiar and unfamiliar route descriptions. In addition, choice point landmarks were used for unfamiliar route descriptions effectively. The result on landmarks on route learning is in agreement with the findings of the previous research (Tlauka and Wilson 1994). Hence, these studies indicated that landmarks are effective not only at decision points but also along a route (please see Table 4 for the summary of the mentioned papers).

**Table 4:** Summary table of the studies on location of landmarks. The names are listed based on their appearance in the text

Author	Year	No. of people	Environment		Layout		Task						Saliency			Visibility		Finding
			Real	Virtual	Grid	Non-grid	Wayfinding	Orientation	Route description	Recognition /preference	Sketch	Other (gaze etc.)	Visual	Structural	Cognitive	Global	Local	
Chan et al	2012	Not an experimental study												X				Landmark functions were divided into four: beacon, orientation cue, associative cue, and reference frame. Any environmental object can serve as a landmark, including the boundaries and extended surfaces
Janzen	2006	20–21–23		X	X					X	X			X				Landmarks were recalled better if they were located near decision points. Response time was also shorter for recall task for objects at decision points
Klippel and Winter	2005	Not an experimental study												X				Landmarks at decision points were more effective than landmarks along the route
Röser et al	2012	45–18		X	X					X				X				Landmarks should be located at the side of the intersection on which a turn needs to be executed
Claramunt and Winter	2007		X			X	Not an experimental study (method is adapted to an area only)							X				Researchers could identify structural qualities of places, paths, barriers and districts. These were applied to route guidance
Lovelace et al	1999	31	X			X			X	X				X				Not only landmarks at decision points, but also landmarks on route were used by people
Darken and Sibert	1993	9		X		X	X		X			X		X				Maintaining orientation was a problem when landmarks were non-directional
Cliburn et al	2007	86		X		X	X					X		X				No impact of dynamically placed landmarks on navigation was observed for people unfamiliar with the environment. However, dynamically placed landmarks that remained between visits were used by people
Von Stülpnagel et al	2014	115–53		X		X (grid-like)	X				X	X		X				No impact of individual landmark placement on wayfinding performance was observed

In addition to the studies about landmarks at decision points and landmarks on-route, various studies explored dynamically placed landmarks in order to better observe the effective placements of landmarks. Darken and Sibert (1993) aimed to investigate the design principles for navigational aids in VEs. They presented different scenarios such as breadcrumbs or map view scenario (subjects could consult the map at will), and subjects’ wayfinding behavior was observed. ‘Breadcrumbs’ (or the Hansel and Gretel Scenario) was a manual landmarking technique where subjects would mark their position with an object. Accordingly, landmarks can either be dropped at regular intervals along a straight line between two positions to mark places or they can be dropped to be used as directional indicators. The researchers mentioned that if dynamically placed landmarks are also directional, they would make it easier to follow a path. Cliburn and his colleagues (2007) analyzed four conditions in VEs: virtual environments with no landmarks, with statically placed landmarks (objects were located at the intersections), landmarks dynamically placed at the subject's discretion that disappeared from trial to trial and landmarks dynamically placed that remained from trial to trial. They asked participants to navigate in an environment multiple times and they introduced two hypotheses for this study; (1) dynamically placed landmarks can be effective for first-time searchers, and (2) dynamically placed landmarks, which remain between visits, can also be beneficial. They observed that subjects traveled further distances during their first trial in all conditions. Therefore, they could not support their first hypothesis; however, they observed that dynamically placed landmarks, which remain between trials, could be effective on wayfinding. Participants traveled longer distances when there were no landmarks, compared to the other three conditions. Hence, it can be argued that all three conditions helped people to complete the task in a shorter time compared to no landmark condition. It is also discovered that different strategies were used to drop the landmarks, including indicating the locations of spheres.

Similarly, Von Stülpnagel et al. (2014) were interested in the impacts of navigator-driven and individual landmark-placement on spatial learning. They created three different conditions: individual landmark condition (placing up to four objects), preplaced landmark condition (four landmarks were replaced so that at least one of them would be visible from any point) and no landmark condition. The researchers developed three VEs with simple geometric shapes and asked the participants to explore the environments and draw a sketch map. As a second study, participants were asked to explore a virtual model of Tate Gallery and to find three goal locations. This time the researchers used non-directional and directional landmarks. They analyzed both the mean time and mean distance of wayfinding performance. They observed that participants tended to place landmarks at the most central and visible locations. On the other hand, they could not find any advantages of individual landmark placement for wayfinding performance. Hence, the impact of dynamically placed landmarks can still be debated. However, it can be concluded that for dynamically placed landmarks, the visibility of objects is the most important.

#### Personal and emotional landmarks

Personal dimensions of landmarks were also discussed in landmark studies as well as emotional landmarks. Many factors including but not limited to people’s demographic information, interests or emotions that are evoked by an environment (or a landmark) can also affect wayfinding procedure, which can be considered as a part of cognitive saliency.

To identify personal landmarks, various measures were developed. Nuhn (2020) proposed that personal dimensions of landmarks can be analyzed using various measures: spatial knowledge (no knowledge, landmark, route or survey knowledge), personal goals (known goals, new goals or exploratory), personal interests such as cultural, historical etc., personal background (for example, education, age or cultural background), individual traits that can be determined through psychological tests or other personal dimensions (positive, negative or neutral landmarks). Götze and Boye (2016) used a methodology that automatically derived salience using route instructions collected from subjects. They asked subjects to walk a specific route and describe the path. People’s verbal descriptions were recorded and transcribed. Each segment of the path was annotated with landmarks that participants referred to. Then people’s personal salience model was derived with an algorithm, which determined the most appropriate landmarks to refer in new situations. This approach is quite unique and significant as it produces personal results. However, as the model is user-related, it is necessary to include a large number of participants to have more accurate results. Nuhn and Timpf (2017) also proposed a multidimensional model for the selection of personalized landmarks and extended existing approaches by using the personal dimensions of landmarks (Nuhn and Timpf 2018). The underlying idea was that landmark selection could vary for individuals. Hence, in the former, non-experimental study (Nuhn and Timpf 2017), they aimed to understand the factors that might affect people’s landmark selections, focusing on people’s personal interests, goals and backgrounds. They argued that personal interests could be closely related to different functions (e.g., art, gastronomy, etc.) and could be obtained with the usage of sensors in smartphones. Personal goals were categorized under three headings: known goals (reaching a known, particular location), new goals (reaching a different, new location-probably with the help of a wayfinding aid) and exploratory travel (traveling without having a specific goal location) (Golledge 1999; Wiener et al. 2009). The researchers suggested that personal background could be analyzed by checking the gender, age and country of residence information. This study provided detailed explanation of how cognitive saliency can be measured and different categories that can be used. Even though these definitions are important for the selection of different landmarks or different routes, it was not clear how to adapt these classifications, fast and efficiently, to a wayfinding task. In the latter—again, non-experimental—study (Nuhn and Timpf 2018), authors extended earlier research on landmark saliency (Raubal and Winter 2002) by using their definitions. In addition to the visual, structural and semantic landmarks, the researchers defined descriptive and environmental landmarks. Accordingly, descriptive landmarks were defined with the explicit marks (signs etc. so that one can use these information in wayfinding tasks) and the number of words (long descriptions can cause confusion so the objects that might need a longer explanation can be excluded from a task) while environmental attributes were defined, in advance, through their visibility, orientation, distance and uniqueness. They considered travelers’ interest, education, country of residence and background and an overall saliency was described using these variables. It was observed that varying attribute values for the personal dimension changed the most salient landmark. This study was another good attempt in identifying landmark saliency as it extended the existing literature by using semantic and environmental attributes. These two papers were essential as they provided personalized categories to better understand landmarks. However, the implementation of these measures to actual environments is still a challenge (please see Table 5 for the summary of the mentioned papers).

**Table 5:** Summary table of the studies on personal and emotional landmarks. The names are listed based on their appearance in the text

Author	Year	No. of people	Environment		Layout		Task						Saliency			Visibility		Finding
			Real	Virtual	Grid	Non-grid	Wayfinding	Orientation	Route description	Recognition /preference	Sketch	Other (gaze etc.)	Visual	Structural	Cognitive	Global	Local	
Nuhn	2020	51	X			X				X			X	X	X			The personalized model did not identify significantly more landmarks than a conventional model. Hence, collecting personal information was unlikely to justify the effort
Götze and Boye	2016	10	X			X			X						X			Individual salience models were developed so that the landmark(s) that are most appropriate to refer to in new situations are determined
Nuhn and Timpf	2017	Not an experimental study													X			People’s spatial knowledge, interests, goals and background were considered while explaining personal landmarks
Nuhn and Timpf	2018	Not an experimental study											X	X	X			Personal dimensions had an impact on which landmarks were considered the most salient
Kattenbeck	2016	112	X							X			X	X	X			It is stated that important subdimensions of salience are missing in current theories and both emotional salience and familiarity were identified as two important measures
Kattenbeck et al	2018	112–109	X							X			X	X	X			Personal dimensions had an important impact on saliency
Ruotolo et al	2019	150		X		X				X	X	X			X			Landmarks with a positive value were better remembered than landmarks with neutral or negative value
Palmiero and Piccardi	2017	75	X			X				X	X				X			Both positive and negative landmarks facilitated learning the path compared to neutral landmarks
Piccardi et al	2020	115	X			X				X	X				X			Positive and negative landmarks enhanced the task of learning the path. Only positive landmarks enhanced the reproduction of the map
Balaban et al	2017	24–24		X						X					X			Landmarks associated with negative emotional responses had a positive effect on the results

Other studies also examined salient landmarks and identified overall salience using visual, structural and cognitive salience as well as the degree of prototypicality (examines how typical an object is) and advance visibility (Kattenbeck 2016; Kattenbeck et al. 2018). In these two studies, Kattenbeck et al. designed a survey-based salience model to understand whether weights used in saliency studies are robust across environments, objects and observers. For this purpose, the researchers designed surveys, asked people to rate objects and identify objects along the route. Participants’ gender and sense of direction were also considered. The research is conducted in one of the cities in Germany (Kattenbeck 2016) and then extended and included another city for comparison (Kattenbeck et al. 2018). They discovered that personal dimensions have an important impact on the results. For instance, they observed that visual landmarks have a higher impact on overall salience for females than males, or a poorer orientation in females yields a higher importance of visual salience than is the case for good orientation in women. The researchers stated that more studies on salience and especially on personal landmarks are needed. These studies also focused on personal dimensions of landmarks and highlighted their significance.

It has also been discussed that seeing pleasant or threatening objects (emotional salience of objects) can affect our wayfinding performance. Ruotolo et al. (2019) assessed how people memorize spatial information of emotionally laden landmarks along a route. Three groups of participants were asked to watch a movie of a virtual walk and the route contained either positive, negative or neutral landmarks. Then they were asked to recognize the landmarks, indicate position of landmarks along the route, judge the length of the route and draw the route. The results showed that participants who saw positive landmarks were more accurate in locating the landmarks and drawing the route. Palmiero and Piccardi (2017) asked people to learn and retain an eight-square path with positive, negative or neutral landmarks. Participants’ ability to learn the path, recall and reproduce the path was tested. The results indicated that both positive and negative emotional landmarks equally enhanced the learning of the path. Additionally, positive landmarks improved the reproduction of the path more than negative or neutral landmarks. Piccardi et al. (2020) created five groups using high arousal and positive landmarks, low arousal and positive landmarks, high arousal and negative landmarks, low arousal and negative landmarks and neutral landmarks. Five groups of participants were asked to learn a path and recall it after five minutes, track the learnt path and recognize landmarks. No differences due to landmarks were found in the recall task (after 5 min.). On the other hand, it was observed that people who saw positive or negative landmarks were faster in learning the path and people exposed to negative/high arousal landmarks were less successful in drawing the path than the other groups. However, results of another study (Balaban et al. 2017), in which people were asked to watch a movie of a virtual route and then indicate the correct turn for each decision point, highlighted that the participants remembered negative landmarks better and performed more accurately with negative landmarks than with positive or neutral landmarks. Hence, these studies discovered that the emotional impacts of landmark may also shape people’s wayfinding performance.

#### Gaze behavior and saliency

Other studies investigated gaze behavior and tried to explain how wayfinding process is affected from where people look at or the relationship between eye-tracking data and landmark selections. An important study was conducted by Viaene et al. (2014). They aimed to detect indoor landmarks by using eye-tracking data. They asked people to explore a complex university building twice: once with the experimenter and once by themselves. Participants’ eye-tracking data were recorded during the task; participants were then asked to verbalize everything related to the navigational task and the building. The fixated objects and the mentioned objects were then compared. In total, 41% of the verbalization referred to a potential landmark and 69% of the mentioned potential structural and object landmarks were fixated on. Hence, the researchers stated that eye tracking could provide qualitative and complete data to identify indoor landmarks. Wiener et al. (2012) aimed to understand the relationship between spatial decision making and gaze behavior as well as the ways to analyze geometric features to predict gaze behavior. They asked participants to search for an object that was placed in a virtual environment. Participants were asked to look at a series of images and choose left or right at the decision points. Their eye movements were recorded during the experiment. After rotating the images, participants were asked to look at the images again. Results of this study showed that people have a tendency to choose paths containing longer lines of sight, which again shows the effect of visibility on people’s behavior. In addition, researchers stated that local geometric features and changes in the geometry had an influence on where people looked at. More specifically, corners, openings and occlusions had a strong predictive power on where people looked at. However, the researchers also found that changes in geometry were good predictors for fixation but weak predictors for movement decisions whereas lines of sight were good predictors for movement decisions but weak predictors for fixations. Therefore, depending on the task, results varied in this study, which can be further explored. In another study, the researchers were interested in associating the positions of decision-related information with the placement of the actual choices (Wiener et al. 2011). In the training phase, participants were passively transported along a route in a virtual environment that consisted of 18 decision points. Both unique and non-unique landmarks occurred at each intersection. In the test phase, participants were asked to select the direction (left or right) using static images. Their performance (correct choices), response time and gaze behavior were recorded. In this study, unique landmarks were images that were present only once, and non-unique landmarks were always the image of a pig. An increase in reaction times was discovered when unique landmarks were not on the required movement direction. This result also shows the impact of structural salience of landmarks on decision making, which could be detected by eye-fixations (please see Table 6 for the summary of the mentioned papers).

**Table 6:** Summary table of the studies on gaze behavior. The names are listed based on their appearance in the text

Author	Year	No. of people	Environment	Layout			Task						Saliency			Visibility		Finding
			Real	Virtual	Grid	Non-grid	Wayfinding	Orientation	Route description	Recognition /preference	Sketch	Other (gaze etc.)	Visual	Structural	Cognitive	Global	Local	
Viaene et al	2014	9	X				X		X			X	X	X				Participants' descriptions were mostly in line with their eye-tracking data mostly (69%)
Wiener et al	2012	20–20–16–18		X		X				X		X	X	X				Gaze during a wayfinding task could be predicted by a subset of environmental features
Wiener et al	2011	17		X						X		X	X	X				An increase was discovered in reaction times when unique landmarks were not on the required movement direction
Kiefer et al	2014	10–15	X			X				X		X	X					Successful participants focused their attention significantly more on the helpful map symbols. Successful and unsuccessful landmarks based on self-localization could be estimated by considering gaze patterns
Wenczel et al	2017	23	X			X	X		X	X		X	X	X				The intention to learn a route led to an increased focus on more structurally salient landmarks. In addition, visually salient objects led to longer fixation times

Kiefer et al. (2014), on the other hand, aimed to observe the visual matching process between environment and the map during self-localization. Two experiments were organized. In the first experiment, participants judged whether or not an iconic map was a correct representation of an environment they were shown. Participants, who were unfamiliar with Zurich (Switzerland), took part in the study and ten people’s results were used. In the second experiment, people were asked to mark their position (a square in Zurich) on a tourist map. In both maps, participants could see various landmarks that could help them identify the environment, and in both studies their eye movements were recorded. Results showed that participants, who completed the tasks successfully, focused their attention significantly more on the helpful symbols. In addition, it was discovered that successful and unsuccessful landmark-based localization could be differentiated with respect to fixation distributions and sequences of visual attention. Wenczel et al. (2017) aimed to understand the extent to which the selection of landmarks could be established from eye tracking. Participants were asked to explore two routes and provide route descriptions. They were then instructed to walk both routes from memory. They were also asked to indicate the landmarks on their route. The authors estimated visual saliency by using the eye-tracking system (the results were rated by two raters on a 5-point scale from visually non-salient to visually very salient). Structural saliency was analyzed as landmarks at potential choice points, at choice points and on route. The results showed that participants focused most on the corners in the turning direction and gaze patterns were affected by direct line of sight, with landmarks at the most visible corners. Therefore, the findings highlighted that learning a route is related with structurally salient landmarks. In addition, the researchers discovered that visually salient objects led to longer fixation times. However, this effect was unaltered by learning intention. Here, the approach to understand salient landmarks by directly using eye-tracking data was introduced, which was employed to explore visual landmarks. A review article (Kiefer et al. 2017) listed several advantages of using visual attention systems, which included the theory that wayfinding aids rely on visual information and that it is easier to measure visual attention than other senses. Hence, fixations can provide information on people’s wayfinding choices and landmark selections. Even though it is still hard to process eye-tracking data, this area can be further explored.

### Findings of different studies on saliency of landmarks

In the “Measures used in explaining saliency” section, we discussed various measures used in explaining saliency. In this section, we focus on the results of saliency studies in which researchers aimed to define the most effective saliency criteria (visual/ structural/ cognitive) for wayfinding.

The findings of the studies on saliency, however, vary. First, Peters et al. (2010) aimed to use an automatic landmark selection tool, to identify landmarks and to compare the results with people’s landmark choices and behavioral characteristics. They used a virtual environment with box-like buildings where there were two alternative routes (a short route with 8 intersections and 38 facades; and a longer route with 16 intersections and 126 facades). Students participated in the experiment and they actively navigated through the virtual environment using a joystick. All participants experienced the environments and wrote route descriptions. Structural, semantic saliency, visibility and visual saliency (based on color) scores of landmarks were computed. The researchers discovered that the highest, positive correlations between saliency scores and people’s choices were observed for advance visibility and structural salience. Hence, they claimed that structural salience and advance visibility had higher impacts on people’s landmark choices. Winter et al. (2005) theorized that landmarks are not global features but are more personal. They conducted a human subject test and focused on people’s landmark selections during the daytime and night-time. After being asked for demographic information as well as their familiarity with the area, participants were shown 4 panoramic images of Vienna (a street image with various buildings). Half of the participants were shown daylight images while the other half were shown the night-time images, then they were asked to choose a prominent façade. In the second part of the study, the same participants were asked to rank characteristics of the facades so that the researchers could understand which characteristics of landmarks made them more noticeable. The authors discovered that several facades were ranked significantly different between daytime and night-time images. More importantly, it was observed that compared to the area, shape or marks (e.g., signs) information, visibility was the most effective factor for an object to become a salient one.

In their study, Miller and Carlson (2011) also aimed to determine the factors that make a landmark salient. They used Caduff and Timpf’s definition (2008) and focused on perceptual and contextual characteristics of landmarks in a virtual museum. For perceptual characteristics, they focused on color and size, and for contextual characteristic they focused on objects at decision points with a turn, without a turn and at non-decision points. Participants were asked to learn a route and memorize the objects they saw. They were then asked to give directions of the route, draw a map with instructions and recognize whether or not an object they were shown was in the museum. The findings of this study suggested that both perceptual and contextual landmarks are important components of objects which are deemed salient. Von Stülpnagel and Frankenstein (2015) aimed to examine the effect of configurational salience of global and local landmarks in producing sketch maps as compared to visual salience. A virtual environment was created for this study. The environment consisted of 20 global landmarks (larger geographic features such as buildings) and 48 local landmarks (such as cars). People were asked to complete sketch (a group produced maps while exploring the environment), map (second group explored the environment with a map) or free (the third group explored the environment without additional aids) condition. The authors discovered that people do not only rely on visually salient objects, but they also use configurationally (or structurally) salient ones for orientation. Participants sketched large, visible and integrated landmarks more for the tasks and used global landmarks more frequently when these objects had a different size compared to their surroundings. Local landmarks were used more when they were visually salient and visible (isovist size was larger). Moreover, it was observed that in sketch condition, participants added more landmarks compared to map and free conditions (they drew maps from memory). The differences in the task, therefore, affected people’s observations in this study (similar to the findings of studies on visibility of landmarks). Therefore, the researchers argued that it would be important to compare the same people’s drawings under different conditions. Results of the above-mentioned studies mainly highlight the impact of the structural as well as visual characteristics (color, visibility) of landmarks during wayfinding.

The same virtual environment, Squareland, was used in another study, where the researchers aimed to understand the impact of famous landmarks and to determine the changes in wayfinding performances with visual, verbal and acoustic cues (Hamburger and Röser 2014). In the first experiment, university students were randomly assigned to one of the three conditions (visual, verbal, acoustic). In the recognition task, participants indicated whether they saw an image or word (of an animal) or heard animal sounds. Then they were re-shown the environment and were asked which way to move at each intersection. The researchers discovered that verbal and sound instructions were better remembered than pictures. In wayfinding task, however, they did not find any significant differences. Therefore, the researchers concluded that further psychological research needs be conducted to better understand the underlying reasons of these differences. In the second experiment, 20 students were asked to view visually salient but unfamiliar buildings as well as visually salient and familiar buildings in the same environment. The findings indicated that famous buildings (visually familiar) were better recognized by people.

Stankiewicz and Kalia (2007), on the other hand, conducted three experiments to investigate the effects of structural and object (statues presented within the virtual environment) landmarks in VEs. In all experiments, participants were asked to navigate in the environment and answer landmark queries. The results of this study showed that humans tend to remember structural landmarks more than object landmarks, and even if the information content of object landmarks was greater than that of structural landmark, participants’ memory recall for the two types of landmarks was still the same. This study also highlighted the significance of structural saliency.

Another research study was conducted to understand the significance of semantic landmarks in both familiar and unfamiliar environments (Quesnot and Roche 2015b). Individuals familiar and unfamiliar with Quebec city (Canada) participated in an online study. After answering questions about their demographic information and familiarity with the city, the participants were shown different parts of the city and were asked to choose two landmarks between a set of four potential landmarks for each intersection. Visual, structural and semantic saliency scores for landmarks were also computed. The study showed that visual salience was closely related to people’s preferences. Furthermore, the results of this study showed that people, who were familiar with the environment, focused on semantic landmarks regardless of a low visual salience. People, who were unfamiliar with the environment, on the other hand, relied on highly visible landmarks. This study, therefore, is an important example of how familiarity affects people’s landmark choices (similar findings were discussed for the visibility of landmarks, please see Table 7 for the summary of the mentioned papers).

**Table 7:** Summary table of the studies on landmark saliency. The names are listed based on their appearance in the text

Author	Year	No. of people	Environment		Layout		Task						Saliency			Visibility		Finding
			Real	Virtual	Grid	Non-grid	Wayfinding	Orientation	Route description	Recognition /preference	Sketch	Other (gaze etc.)	Visual	Structural	Cognitive	Global	Local	
Peters et al	2010	24		X		X (grid-like)	X		X			X	X	X	X			Visibility and structural salience had higher positive correlations with the measured performance. Hence, they have higher impact on people's landmark selection
Winter et al	2005	92	X							X			X		X			Visibility was the most prominent factor
Miller and Carlson	2011	96–32		X	X				X	X	X		X	X				Perceptually and structurally more salient landmarks were recognized faster and were included in the directions and maps
Von Stülpnagel and Frankenstein	2015	45		X							X		X	X		X	X	Participants used both visually and structurally salient landmarks. Large, visible and accessible objects were preferred more compared to sketch
Hamburger and Röser	2014	30–20		X	X		X			X			X		X			Cognitively salient landmarks were better remembered by people. Different modalities did not cause significant changes in wayfinding task
Stankiewicz and Kalia	2007	8–6–4		X	X		X			X			X	X				People were sensitive to the information content of landmarks and inclined to learn structural landmarks better than object landmarks
Quesnot and Roche	2015b	80–63		X	X					X			X	X	X			People familiar with the environment focused on cognitive landmarks regardless of a low visual saliency. People unfamiliar with the environment focused on highly visible landmarks
Albrecht and Von Stülpnagel	2018	34		X	X					X			X	X				People remembered a turn correctly if visually salient landmarks are positioned in the turning direction
Michon and Denis	2001	20–20	X			X							X	X				Visual landmarks were mentioned more frequently when they were close to critical nodes

Another group of research, on the other hand, focused on the impact of combined characteristics of landmarks. Albrecht and Von Stülpnagel (2018) stated that even though visual and structural landmarks supported wayfinding, the interaction between the salience forms has received little attention. Therefore, they aimed to understand the combined effect of visual and structural salience on wayfinding behavior. They located visually salient objects both at structurally salient locations and structurally less salient locations. They used a virtual environment where the intersections were symmetrical and consisted of four buildings located at the four corners. One building was colored differently (visual saliency), and the turning direction was indicated by a yellow line. Participants were first asked to study pictures of the intersections with a given turning direction. They were then presented with one intersection without an indicated turning direction and were asked to make decisions. It was discovered that people tended to remember a turn correctly if a visually salient landmark is located in the turning direction. A similar study was undertaken by Michon and Denis (2001) who asked people unfamiliar with the environment to learn two routes in Paris by navigation and to generate route directions. The authors observed that visual landmarks are better remembered when they are close to nodes. So, for instance, a landmark can be visually salient, but if it is located in a structurally less salient position, then it might be used by less people in wayfinding tasks. This body of literature is also important to understand the combined effect of landmarks, rather than focusing on only one or more characteristics separately (Yesiltepe et al. 2020b). Hence, it can be argued that different characteristics of landmarks are influential for them to become “salient.”

### Summary of the saliency of landmarks

Studies on saliency already discovered some important characteristics of landmarks. Accordingly, visual, structural and cognitive (or semantic) characteristics of landmarks should be considered while measuring saliency of objects. Various measures were developed to analyze saliency of landmarks, including but not limited to area, shape, color, visibility (visual landmarks); people’s interests, background, education, age, gender, the explicit marks of buildings, social media results, emotional landmarks (cognitive landmarks); and the nodes, boundaries, paths, and order of buildings (structural salience). Therefore, it is important for futures studies to integrate various measures to analyze saliency in order to predict people’s choices. Various studies suggest that structural saliency and visual saliency were effective to understand people’s preferences. However, as different measures were developed to understand semantic salience, it is expected that semantic salience would also be effective on understanding people’s behavior.

Literature on location of landmarks (structurally salient landmarks) has some consensus: the existing research highlighted that landmarks are more influential when they are on-route and when they are at decision points with a turn. A route can be defined by using landmarks on route (but not necessarily at decision points). Hence, for instance, we can say “Pass the opera house” and reassure the traveler to continue along the same route. We also can give another instruction and say, “turn right after the opera house and go straight.” This way the traveler will know a change of direction is needed. However, it can be indicated that if a landmark is located on-route and at a decision point, then it can be used by more people during wayfinding. Results of studies on dynamically placed landmarks, on the other hand, varied. More research needs to be carried out in VEs to explore where dynamically placed landmarks are likely to be located. It might also be useful for these studies to compare the impact of directional and non-directional landmarks.

Studies also explored the impact of combining landmark types. More specifically, they discovered that visual objects could be more effective for navigation when they are also structurally salient. However, more research needs to be conducted in this field to better understand the interaction between different saliency measures (for instance, the positions of visually salient landmarks can be changed to determine whether people’s landmark selection preferences also change).

Moreover, current research shows that eye tracking could be an effective way of understanding people’s landmark selections. It is now possible to use eye-tracking data to measure the visual characteristics of landmarks, since people tend to fixate more on salient objects. The most important challenge here is that it is hard to process eye-tracking data due to the increased analysis-time required. However, this is a new and an accurate way of understanding the saliency of objects. Therefore, we believe that further research should be conducted to explore the relationship between eye-fixations and route/landmark choices.

All above-mentioned studies on saliency show that landmark selection is an important area in research. It is important to note that while creating a landmark detection system, it is important to consider (1) reproducibility of models—is it easy to follow the defined steps, are all measures explained clearly?; (2) reproducibility of models in different scales—cost and effort predictions will be important (Richter 2013); (3) whether or not the models will work in practice—not only identify the automatic selection tools but also adapt them to different environments and compare the results with people’s choices (if possible) (Richter 2013, 2017). As people might choose different landmarks for different conditions, automatic detection tools can provide different alternatives for daytime or night-time; or for pedestrians and cars, leading to improved tools. Cost estimations should also be considered while developing new results. In addition, the models that will be developed should also account for the above-mentioned measures to better estimate saliency. It is important for future research to focus on all aspects of salience–visual, structural and semantic (cognitive)—through above-described measures. Even though visual and structural salience are covered by many measures, most of the existing studies on landmark selection failed to measure semantic salience through different measures. Not only the function of buildings but additional components that might affect saliency scores (e.g., people’s cultural background, gender, interests or social media activities etc.) should be adapted in future studies. Furthermore, in addition to buildings, all types of landmark candidates, such as stairs (e.g., The Spanish Stairs in Rome), fountains (e.g., Piazza Navona in Rome), statues (Statue of Liberty in New York) or even urban furniture (e.g., telephone boxes in London), should be adapted to these algorithms since they could have high saliency scores and hence could be used in route-descriptions or for other purposes. In addition, even though there are many automatic detection alternatives, there are currently a limited number of online tools. Hence, online and more user-friendly tools, which provide solutions for many issues mentioned here, need to be developed. Finally, it is also important for models to be tested both by experts—to identify possible problems—and non-experts, to understand whether or not the tools developed are easy enough for everyday use. If all these issues can be resolved, we believe that successful models can be produced and adapted to everyday life wayfinding tasks.

Two more issues can be highlighted for future methodologies. Studies mentioned here used both real and virtual environments as well as one or multiple environments. However, we cannot assert that results vary in different environments. In the previous section (Visibility of landmarks), we saw that environment-familiarity could affect people’s landmark choices (e.g., familiar people tend to use local landmarks). In this section, it was observed that familiarity with the environment also affected people’s saliency evaluations (cognitive salience). Research showed that people who knew an area could rely on semantic landmarks while those unfamiliar with the area relied more on visual landmarks. Hence, environmental familiarity has an impact on landmark evaluations. The type of the task also impacts the findings. Studies mentioned that landmark detection might differ depending on the task people are undertaking or duration of the travel (Sadeghian and Kantardzic 2008). The number of landmarks people remember (Von Stülpnagel and Frankenstein 2015) can be affected by the specific task; and different landmark types could be more helpful for different tasks (Hamburger and Röser 2014; Wiener et al. 2012). Hence, it is important to design experiments based on both the tasks and the landmarks being used.

## Conclusion

In this study, we aimed to understand the characteristics of landmarks used in wayfinding tasks. We asked two questions:

1. Which characteristics of landmarks make them more effective during a wayfinding task? What consensuses are in the literature?
2. What are the gaps in the current literature? And how might these be addressed to better understand the relationship between environments, landmarks and people?

In the light of the first question, we showed that there were consensuses in the literature on different aspects of landmarks: it was observed that some terms such as the *unique* (*distinctive)*, *identifiable* characteristics of landmarks were repeated by authors. Landmarks were identified as salient and communicable objects in an environment that helped people to find their way, orient themselves or describe a route. Past studies clarified that landmark’s specific characteristics allowed them to be selected by more people and because of these characteristics they became unique or identifiable. We investigated these characteristics under two headings in this review: *visibility* and *saliency* of landmarks. For the *saliency of landmarks*, various papers mentioned the visual, structural and cognitive (semantic) characteristics of landmarks. We can state that the first consensus in saliency papers is these three categories. Accordingly, objects should be visually (their color, area, shape or visibility), or structurally (located at easily accessible points), or cognitively (they should be historically or culturally meaningful) distinctive so that they can be used during wayfinding tasks. Structural and visual characteristics were analyzed by various researchers, who concluded that both characteristics had an effect on people’s wayfinding behavior. For visual characteristics, visibility and color were mentioned in several studies. Hence, we can assume that these characteristics are the key for landmarks to be used by people. For the location of landmarks, it was seen that compared to off-route landmarks, landmarks on-routes as well as landmarks at decision points could be remembered more easily by people. More specifically, studies mentioned that decision points, where the direction is changed, can be the ideal locations for landmarks to be selected by a higher number of people.

To answer the second question, on the other hand, it is possible to state that there are numerous gaps in the literature, which can be addressed in future research. The first gap concerns the *saliency of landmarks*. Various studies explored saliency and proposed different measures (e.g., social media, usage of personal choices, people’s demographic data, etc. for semantic salience; nodes, barriers, etc. for structural salience; and visibility, color, area, etc. for visual saliency). These measures are useful to better understand how people choose objects in different virtual or real environments. However, the papers examined in this study showed that the use of measures in investigation of saliency is still limited. Especially cognitive saliency was analyzed by a limited number of measures in studies calculating overall saliency, while many personal or emotional measures were proposed by the researchers. This is one of the significant gaps in the literature. Future research needs to focus on the defined measures and future experiments need to include as many of them as possible to derive accurate results.

Another gap persists in the definition of the *visibility of landmarks*. Research suggests that landmarks can be seen either from many points in an environment (Lynch 1960) or from everywhere (Castelli et al. 2008; Lin et al. 2012). Here we believe that global landmarks are objects that can be seen from many points in an environment, but not necessarily from everywhere, as Lynch (1960) also observed. However, at this point we asked another question: if a global landmark is seen from many points in an environment, then how can we differentiate between local and global landmarks? Many existing studies used obvious objects, such as mountains and towers, to define global landmarks. But when exactly does a local landmark stop being “local” and become a “global” landmark? Can we define a threshold for this transition (which suggests more of a continuum of landmarks than previous research might suggest)? Future research could therefore focus on finding an objective threshold for these two definitions.^3^ Moreover, research on global and local landmarks provided various results: a group of researchers mentioned that global landmarks can be more effective on wayfinding tasks while others mentioned either local landmarks or both global and local landmarks can be more effective. Hence, we believe that more research can be conducted on the visibility of landmarks.

A number of papers claimed that landmarks are one of the three components that create spatial knowledge (Siegel and White 1975). Hence, in addition to designing relatively simple layouts (Arthur and Passini 1992; Gärling et al. 1986; Passini 1984), where people will not get confused, it is also important to have:

- global and local landmarks that are distributed within an environment (although not necessarily distributed evenly, as one might expect to see more landmarks in centers),
- landmarks that are also visually, cognitively or structurally salient. This would reduce the number of wayfinding errors that people make. Moreover, it is also possible to create more attractive routes with the aid of landmarks, such as aesthetically appealing landmarks such as different statues, buildings, etc.

These points can be used for the design of empirical studies as well as in urban design and planning fields. Finally, it is important for studies on visibility or saliency of landmarks to consider the impact of the task, environment, layout or familiarity with the environment, since the results of studies vary based on these factors. Factors that might be considered for future research are listed in the following section.

### Future work

There are several issues that might be considered in future work. Existing literature already underlined the impact of visual and structural characteristics on navigation-related experiments. Even though different measures were developed to understand cognitive saliency, research that conducted experiments using these measures is still limited. Hence, we believe that the new tools can be useful in comparing these three components more effectively and in better understanding the impacts of cognitive salience on wayfinding behavior. The combined impact of different saliency measures was also studied by researchers; however, more research still needs be conducted to better understand the relationship between different landmark characteristics.

Studies claimed the varying results of previous studies to be related to the familiarity with the environment (Lynch 1960; Schwering et al. 2014) or to the scale of the environment (Gardony et al. 2011) or to another condition. The impact of different tasks on wayfinding was also mentioned in studies on the visibility of landmarks (Schwering et al. 2013,2014,2017) and saliency of landmarks (Hamburger and Röser 2014; Sadeghian and Kantardzic 2008; Von Stülpnagel and Frankenstein 2015; Wiener et al. 2012). Landmarks were used during wayfinding for route definition, orientation, recognition or finding a specific location. Findings of previous studies suggested that different types of landmarks can be used for different purposes during wayfinding. For example, global landmarks can be preferred more in spatial orientation or landmarks at decision points can be remembered better in a recognition task or local landmarks can be used more in verbal descriptions. Hence, we can assert that landmark selection is a task-dependent activity. Moreover, varying results are also related to the layout of the environment. It was mentioned that in a gridal network (e.g., North American street networks) with numbered streets and blocks, people might use street names in their route descriptions whereas in Asian cities with relatively organic street layout, people might rely on landmarks (Duckham et al. 2010). It was also mentioned that different layouts could improve route learning (Evans et al. 1984) or in a linear environment people could rely on one type of landmark (Meilinger et al. 2015). Therefore, future research needs to use these different conditions (task, familiarity and layout) and create comparative experiments to better understand the impact of different conditions on landmark usage.

In this research, we focused on the “environment” and “people” in order to identify the criteria that make a landmark “salient.” This review of relevant papers indicated that both of these factors have an effect on landmark salience. Therefore, we can claim that landmark selection is shaped by a multitude of factors, all of which should be taken into consideration to better understand the underlying reasons of landmark selection process. Hence, it is important to highlight that the research in this field can be further investigated by multidisciplinary research groups.

This study does contribute to the literature by analyzing many of the key papers on the impact of landmarks in wayfinding and summarizing their methodology and findings. We believe this paper can be useful for researchers/academics, seeking an overview of this literature, as well as for practitioners, aiming to design easily navigable and memorable environments.

## Footnotes

1 Here, general characteristics of landmarks are described. There are landmarks that are not easily communicable and yet used by some people. However, this represents a small subset.

2 In a VGA analysis, navigable spaces are represented with cells/grids and the relationship between cells is analysed.

3 We raise the idea that if a landmark is visible for more than 25% of any randomly generated route through the system, and this can include a landmark that is continuously visible for an entire section of the route or can include the culmination of discrete glimpse of the landmark, then this is considered to be a global landmark (for more information about the threshold, please see (Yesiltepe et al. 2020a)). Below this threshold landmarks would be considered to be local only. The threshold of 25% has been empirically produced from a large dataset of navigational data the discussion of which is beyond the scope of this paper but will be the focus of an upcoming publication.
